# Development and Clinical Evaluation of a Novel *Talaromyces marneffei* Mp1p Lateral Flow Assay for Rapid Diagnosis of Talaromycosis

**DOI:** 10.1093/cid/ciag004

**Published:** 2026-01-06

**Authors:** Nguyen Thi Mai Thu, Lottie Brown, Oriana Hawkins, Konner Bloss, Sruthi Venugopalan, Meredith Brown, Jonathan Fenhaus, Heera Natesan Sambath, Jialin Liu, Lyla Clancy, Jian-Piao Cai, Jasper Fuk-Woo Chan, K Y Yuen, Vo Trieu Ly, Ngo Thi Hoa, Thuy Le

**Affiliations:** Division of Infectious Diseases and International Health, Duke University School of Medicine, Durham, North Carolina, USA; Tropical Medicine Research Center for Talaromycosis, Centre for Biomedical Research, Pham Ngoc Thach University of Medicine, Ho Chi Minh City, Vietnam; Clinical Infection Unit, St George's Hospital, St George's NHS Foundation Trust, London, United Kingdom; School of Health and Medical Sciences, Institute of Infection and Immunity, City St George's University of London, London, United Kingdom; Immuno-Mycologics, Inc. (IMMY), Norman, Oklahoma, USA; Immuno-Mycologics, Inc. (IMMY), Norman, Oklahoma, USA; Department of Internal Medicine, University of Nevada Reno School of Medicine, Reno, Nevada, USA; Immuno-Mycologics, Inc. (IMMY), Norman, Oklahoma, USA; Immuno-Mycologics, Inc. (IMMY), Norman, Oklahoma, USA; Division of Infectious Diseases and International Health, Duke University School of Medicine, Durham, North Carolina, USA; Division of Infectious Diseases and International Health, Duke University School of Medicine, Durham, North Carolina, USA; Division of Infectious Diseases and International Health, Duke University School of Medicine, Durham, North Carolina, USA; State Key Laboratory of Emerging Infectious Diseases, Carol Yu Center for Infection, Department of Microbiology, School of Clinical Medicine, Li Ka Shing Faculty of Medicine, The University of Hong Kong, Pokfulam, Hong Kong Special Administrative Region, China; State Key Laboratory of Emerging Infectious Diseases, Carol Yu Center for Infection, Department of Microbiology, School of Clinical Medicine, Li Ka Shing Faculty of Medicine, The University of Hong Kong, Pokfulam, Hong Kong Special Administrative Region, China; Department of Infectious Diseases and Microbiology, The University of Hong Kong-Shenzhen Hospital, Shenzhen, Guangdong Province, China; State Key Laboratory of Emerging Infectious Diseases, Carol Yu Center for Infection, Department of Microbiology, School of Clinical Medicine, Li Ka Shing Faculty of Medicine, The University of Hong Kong, Pokfulam, Hong Kong Special Administrative Region, China; Department of Infectious Diseases and Microbiology, The University of Hong Kong-Shenzhen Hospital, Shenzhen, Guangdong Province, China; Tropical Medicine Research Center for Talaromycosis, Centre for Biomedical Research, Pham Ngoc Thach University of Medicine, Ho Chi Minh City, Vietnam; Department of Infectious Diseases, Hospital for Tropical Diseases, Ho Chi Minh City, Vietnam; Tropical Medicine Research Center for Talaromycosis, Centre for Biomedical Research, Pham Ngoc Thach University of Medicine, Ho Chi Minh City, Vietnam; Oxford University Clinical Research Unit, Ho Chi Minh City, Vietnam; Nuffield Department of Medicine, University of Oxford, Oxford, United Kingdom; Division of Infectious Diseases and International Health, Duke University School of Medicine, Durham, North Carolina, USA; Tropical Medicine Research Center for Talaromycosis, Centre for Biomedical Research, Pham Ngoc Thach University of Medicine, Ho Chi Minh City, Vietnam

**Keywords:** talaromycosis, diagnosis, *Talaromyces marneffei*, lateral flow assay, Mp1p antigen

## Abstract

**Background:**

The diagnosis of talaromycosis, a life-threatening invasive fungal disease endemic in Southeast Asia, still relies on protracted culture methods. We report the development and clinical evaluation of a novel lateral flow assay (LFA) for the rapid diagnosis of talaromycosis.

**Methods:**

The LFA used 2 novel monoclonal antibodies targeting the *Talaromyces marneffei*-specific protein Mp1p. In a retrospective, case-cohort study, we evaluated the diagnostic performance of the Mp1p LFA against the reference standard of culture-proven talaromycosis from any clinical specimens, and compared to our validated Mp1p enzyme immunoassay (EIA) in paired plasma and urine samples from 239 talaromycosis and 160 non-talaromycosis participants randomly selected from cohorts of hospitalized adults with advanced human immunodeficiency virus (HIV) disease from 5 centers across Vietnam.

**Results:**

The Mp1p LFA demonstrated an analytical limit-of-detection of 400 pg/mL, comparable to the EIA. No cross-reactivity with 16 common human fungal pathogens was observed. Clinical sensitivity was higher in urine compared to plasma (95.4% vs 88.7%, *P* < .01), and clinical specificity was >95% in both specimen types. Clinical performance was similar to the EIA: sensitivity 95.4% versus 97.1%, *P* = .87 and specificity 95.6% versus 97.5%, *P* =1.0. Both the LFA and EIA were substantially more sensitive than conventional blood culture collected at the same time (95.4% vs 97.1% vs 77.8%, *P* < .01).

**Conclusions:**

The Mp1p LFA has excellent diagnostic performance, comparable to the EIA, is superior to blood culture, and has the potential to rapidly rule in and rule out talaromycosis at the point-of-care within 15 minutes without need for laboratory infrastructure.

## Teaser

We developed a lateral flow assay (LFA) targeting *Talaromyces marneffei*-specific protein Mp1p in plasma and urine. The Mp1p LFA demonstrated sensitivity and specificity above 95%, and has the potential to rule in and rule out talaromycosis within 15 minutes at the point-of-care.

## BACKGROUND

Talaromycosis is an invasive fungal disease caused by the thermally dimorphic fungus *Talaromyces marneffei* [1, 2]. Talaromycosis is endemic in Southeast Asia, where it has emerged as a leading cause of mortality among people with advanced human immunodeficiency virus (HIV) disease (AHD) and is increasingly reported in other immunocompromising conditions or structural lung diseases [3–6]. Despite the high mortality on treatment of 11% to 30% [3, 7, 8], talaromycosis has received little programmatic attention and resources, prompting a global call for designation as a neglected tropical disease [4] and inclusion of *T. marneffei* as a World Health Organization's Fungal Priority Pathogen [9, 10]. The current diagnosis still relies on decades-old methods of culturing *T. marneffei* from blood or other clinical specimens, which takes 4–28 days and is insensitive, missing 30%–50% of disease [3, 11, 12].

Antigen detection has emerged as a rapid, accurate, and non-invasive tool for talaromycosis diagnosis [11, 13–22]. Two promising enzyme immunoassays (EIA) (the 4D1 EIA [15] and the Mp1p EIA [16]) have been developed, along with their early point-of-care prototypes (the 4D1 immunochromatographic test [17] and the D4 point-of-care test [18]). Among these, only the Mp1p EIA has been extensively evaluated [13, 16, 19], prospectively validated [23], and is available as a commercial kit by Wantai in China [13]. Unlike the 4D1 EIA which targets a nonspecific cytoplasmic yeast antigen, our Mp1p EIA targets the *T. marneffei*-specific cell wall mannoprotein Mp1p encoded by the *MP1* gene [24]. Mp1p is an important virulence factor of *T. marneffei* [25], highly immunogenic, and secreted in abundance in the blood and urine of patients during infection [24], making it a sensitive and specific target for immunodiagnostic development. With reference to culture-confirmed talaromycosis, our in-house Mp1p EIA demonstrated sensitivity of 88% and specificity of 98% in plasma samples of 372 cases and 517 controls [16] and was superior to blood culture in sensitivity (86% *vs* 73%). However, real time Mp1p EIA testing is labor- and resource-intensive, requiring samples to be sent to a central laboratory with dedicated infrastructure and technical expertise [13]. The World Health Organization (WHO) has identified the lateral flow assay (LFA) as a priority tool to close this diagnostic gap [26], which can be performed in any clinic, hospital, or community health center at the point-of-care, without electricity, specialized equipment or laboratory expertise [27]. Here, we describe the development and clinical evaluation of the Mp1p LFA for the rapid diagnosis of talaromycosis.

## METHODS

### Development of the Mp1p LFA

The development of the Mp1p LFA was led by Immuno-Mycologics, Inc. (IMMY, Norman, Oklahoma, USA) in partnership with the University of Hong Kong. The general principles of the Mp1p LFA are summarized in Figure 1. A set of 20 novel anti-Mp1p monoclonal antibodies were developed, and the 2 most promising antibodies were selected for further development due to their high affinity to Mp1p in the LFA format. Detailed assay development procedures are proprietary to IMMY.

**Figure 1.**
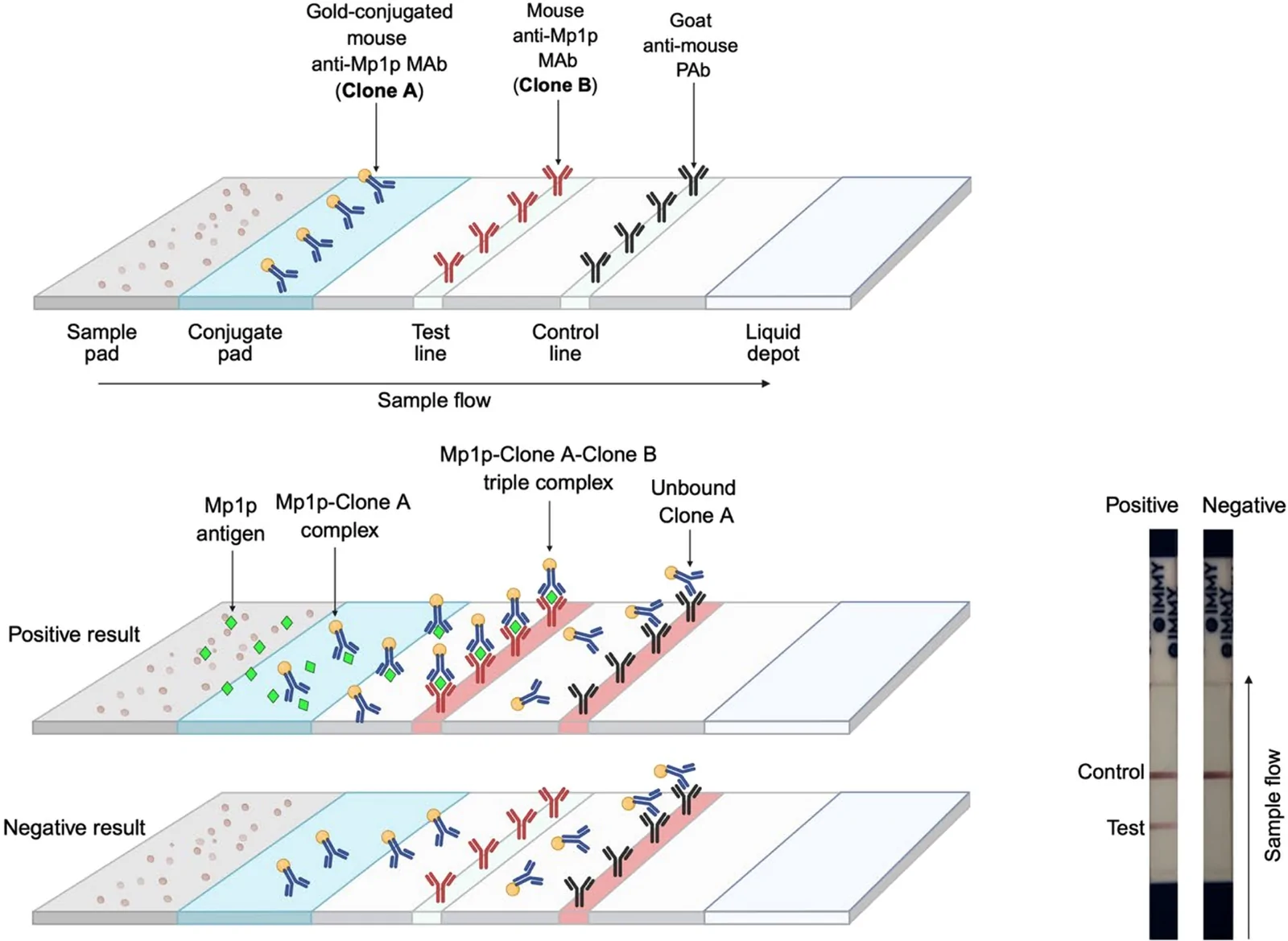
The principle of Mp1p LFA. The development of the Mp1p LFA was led by Immuno-Mycologics, Inc. (IMMY, Norman, Oklahoma, USA) in partnership with the University of Hong Kong (provided access to the original raw materials including Mp1p antigen, protein and antibody sequences, and technical expertise) and Duke University (provided technical and clinical expertise in the study design and implementation). The sample is diluted in sample buffer and added to the integrated sample and conjugate pad. As the sample migrates through the pad by capillary flow, it solubilizes the gold-conjugated anti-Mp1p monoclonal antibody (MAb, Clone A). If Mp1p antigen is present in the sample, Mp1p binds to anti-Mp1p MAb to form an antigen-antibody complex (Mp1p-Clone A complex). The nitrocellulose is coated with an immobilized mouse anti-Mp1p MAb (Clone B). If present, Mp1p-Clone A complex binds to Clone B to produce a triple complex (Mp1p-Clone A-Clone B triple complex) which produces the test line. The control line region is coated with immobilized goat anti-mouse PAb. Unbound gold-conjugated anti-Mp1p MAb (Clone A) binds to goat anti-mouse PAb to produce a control line which confirms proper assay function. Abbreviations: LFA, lateral flow assay; MAb, monoclonal antibody; PAb, polyclonal antibody.

### Sample Preparation and Testing

Plasma was stored at −70°C and urine at −20°C until thawed for testing. LFA procedures are shown in Supplementary Figure 1. Assay result was determined by visual reading of a positive test line; this was the primary output used to assess performance of the LFA. In the clinical evaluation, the LFA test was independently interpreted by visual inspection by 2 double-blinded operators. Disagreements were resolved by a third operator. In addition to visual reading, we used a commercial cube reader (IMMY) to read the results and quantify the test line intensity. The optimal positivity threshold (ie, the Youden index cutoff) of the cube reader was determined by the receiver operating characteristics (ROC) analysis of clinical samples.

### Determination of the Analytical Limit of Detection

Range finding for the limit of detection (LoD) of the LFA was performed using serial 2-fold dilutions (concentration 0–100 000 pg/mL) of level 2 synthetic urine (Bio-rad, Hercules, Calfornia, USA) and plasma from healthy volunteers spiked with recombinant Mp1p (rMp1p) tested in duplicate. The analytical LoD was determined by testing 20 replicates of rMp1p-spiked urine or plasma at concentrations of 400, 500, 600, 800, and 1000 pg/mL and was defined as the lowest concentration demonstrating ≥95% positivity (C_95_) by visual interpretation per Clinical Laboratory Standard Institute (CLSI) guidance [28, 29]. An analytical LoD (C_95_) was also determined for the cube reader using a preliminary end-user-defined cutoff value of ≥5.

### Determination of Analytical Specificity

Analytical specificity was evaluated using 16 medically relevant fungal isolates including *Aspergillus, Cryptococcus,* and *Histoplasma* spp. (Supplementary Table 1) at 10-fold serial dilutions. In addition, plasma and urine from 30 healthy individuals with no history of talaromycosis were tested. Assay results were determined by visual reading.

### Clinical Study Design and Population

For this diagnostic accuracy study, we employed a nested case-cohort study design utilizing samples from randomly selected sub-cohorts of larger prospectively recruited cohorts of patients with culture-confirmed talaromycosis (cases) and patients with similar risk profile without talaromycosis (controls). Talaromycosis reference standard was defined as a compatible clinical syndrome plus *T. marneffei-*positive culture of blood, or other sterile clinical specimens, consistent with EORTC/MSGERC definitions of proven endemic mycoses (Supplementary Figure 2) [30]. Non-talaromycosis controls were hospitalized adults with AHD who had no clinical or microbiological evidence of talaromycosis over a 6-month follow-up period. Clinical samples included in this study had previously undergone EIA testing in real-time. All participants were adults (≥18 years) with AHD (CD4 count <200 cells/mm^3^ or WHO disease stage 3 or 4) recruited from (1) the IVAP clinical trial conducted at 5 centers across Vietnam between 2012 and 2015 [7] and (2) a single-center, prospective talaromycosis screening study in southern Vietnam between 2018 and 2019 [31] (Figure 2). The nested case-cohort study design is highly efficient, allowing testing of only a subset of the entire cohort, yet is robust in ensuring non-biased sampling of a population for whom the test would be applied in real life.

**Figure 2.**
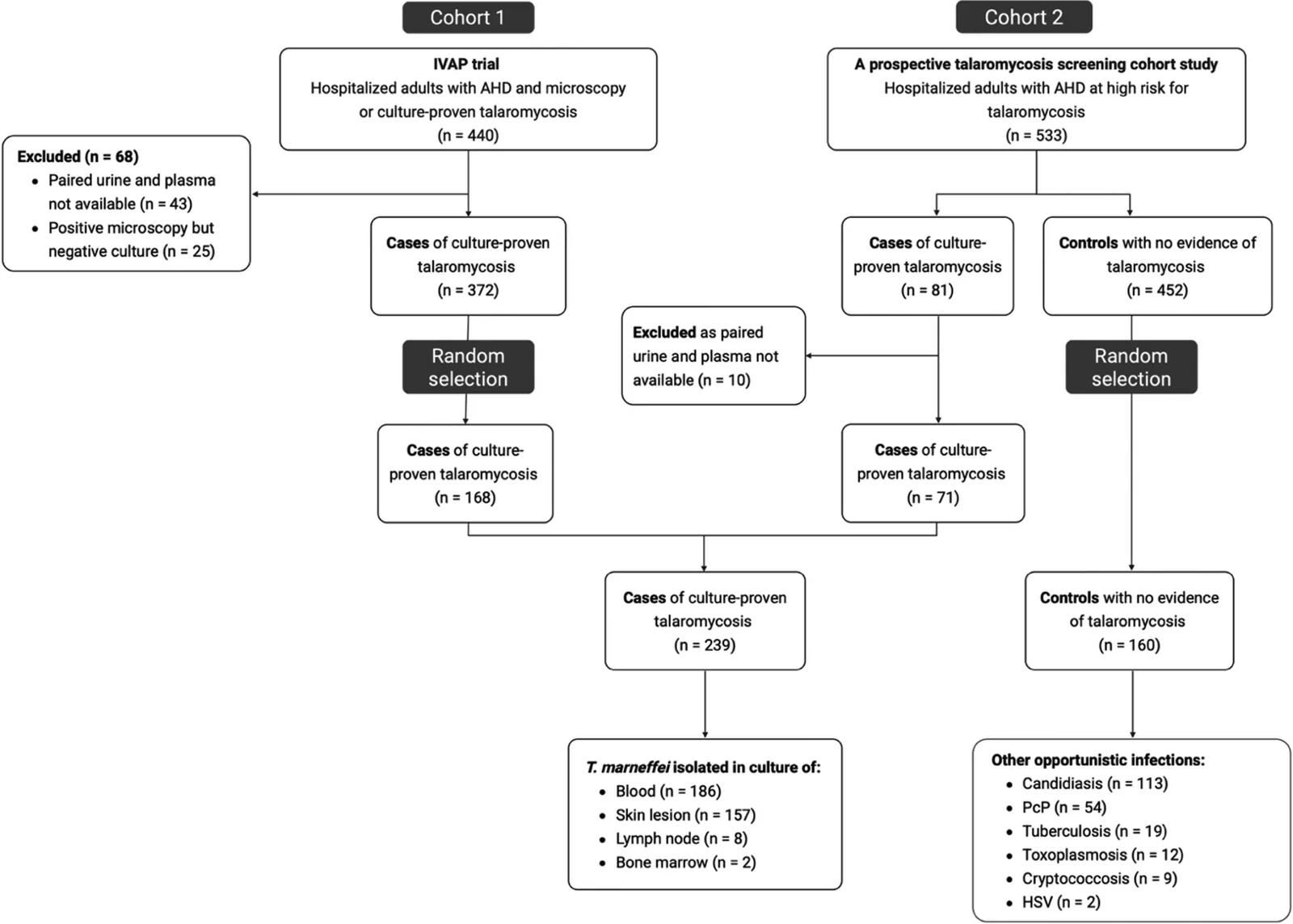
Study population. The selection of case and control participants from their parent cohorts, the clinical specimens used to diagnose talaromycosis, and the other diagnoses of the controls based on STARD guidelines. Cases of culture-proven talaromycosis included 168 participants from the Itraconazole versus Amphotericin for Penicilliosis (IVAP) trial plus 71 participants from the prospective talaromycosis screening cohort study (total N = 239). Controls included 160 participants from the prospective talaromycosis screening study who demonstrated no clinical or culture evidence of talaromycosis over a 6-month follow-up period. Cases and controls were randomly selected from batches of cases and controls based on availability of sample aliquot and volume without knowledge of clinical and laboratory characteristics of patients. Abbreviations: AHD, advanced HIV disease; HSV, herpes simplex virus; n, number; PcP, *Pneumocystis* pneumonia.

### Statistical Analysis

Sample size calculations are presented in Supplementary Figure 3. The diagnostic performance of the LFA in different specimen types was determined based on the reference standard. The primary assay output is based on the visual binary result (yes *vs* no test band), with cube reader reading being the secondary output in our analysis. For the continuous output of the LFA using cube reader, and of the EIA using spectrophotometer, cube reader units and optical density (OD) values were plotted for talaromycosis cases and non-talaromycosis controls by specimen types, and used to construct ROC curves. The discriminative power (ie, the ability to differentiate talaromycosis from non-talaromycosis) for each assay on each specimen type was determined by calculating the area under the ROC curve (AUC). Assay cutoffs were determined based on Youden's index, which maximizes true positives and minimizes false positives. The cutoff values for the LFA and EIA were used to define test positive/negative results. Assay sensitivity, specificity, positive likelihood ratios (LR+), and negative likelihood ratios (LR−), and their corresponding 95% confidence intervals (CI) were calculated for each output of the LFA and EIA.

To compare diagnostic performance of the LFA across three specimen types, the two interpretation methods of the LFA (visual inspection *vs* cube reader), and performance of the three diagnostic modalities (LFA vs EIA vs blood culture), we compared the AUC using DeLong's test and analyzed differences in sensitivity and specificity using Cochran's Q test [32]. When differences reached statistically significance, post hoc pairwise McNemar's test were performed with Bonferroni correction to obtain adjusted *P*-values. Differences in LRs of paired data were compared using a log-linear regression model [33]. In addition, we calculated Cohen's kappa coefficient, as a measure of inter-rater reliability, to evaluate consistency between the two methods of interpretation of the LFA. All statistical analyses were performed using R software version 4.4.2 (R Foundation for Statistical Computing, Vienna, Austria).

## RESULTS

### Characteristics of Study Participants

Figure 2 shows the study flow based on STARD guidelines [34]. A total of 239 cases of culture-confirmed talaromycosis and 166 controls were randomly selected for testing. Cases were diagnosed by positive culture of blood (n = 186), skin lesions (n = 157), lymph nodes (n = 8), bone marrow (n = 2). The characteristics of study participants are shown in Table 1. Cases and controls were similar in median age (33 years vs 34 years, *P* = .07). Among the cases, there were significantly fewer males (67% vs 78%, *P* = .02) and more people who injected drugs (PWID) (21% vs 12%, *P* = .02). Cases had significantly lower CD4 (10 cells/mm^3^ vs 17 cells/mm^3^, *P* < .01). Both cases and controls were diagnosed with a range of other opportunistic infections. Tuberculosis and bacterial sepsis were significantly more common among cases, whereas candidiasis, cryptococcosis and toxoplasmosis were more common among the controls (*P* < .05).

**Table 1.** Characteristics of Study Participants

Characteristics	All (N = 399)	Talaromycosis Cases (n = 239)	Controls Without Talaromycosis (n = 160)	*P* Value
**Demographics**				
Median age in years (IQR)	33 (29–38)	33 (28–37)	34 (29–40)	.07
Male, n (%)	284 (71)	160 (67)	124 (78)	**.02**
PWID, n (%)	70 (18)	51 (21)	36 (12)	**.02**
**Immune status**				
ART status				
Naive	220 (55%)	132 (55%)	88 (55%)	.9
On treatment	179 (45%)	107 (45%)	72 (45%)	
Median CD4 count (cells/µL) (IQR)	13 (5–26)	10 (4–22)	17 (6–36)	**<.01**
Median duration of symptoms (d) (IQR)	17.5 (10–30)	20 (14–30)	14 (10–28)	**<.01**
**Opportunistic infections^a^**				
Tuberculosis, n (%)	57 (18%)	38 (25%)	19 (12%)	**<.01**
Bacterial sepsis, n (%)	7 (2%)	7 (5%)	0 (0%)	**<.01**
CMV, n (%)	2 (1%)	2 (1%)	0 (0%)	.2
HSV, n (%)	10 (3%)	8 (5%)	2 (1%)	.06
PcP, n (%)	92 (29%)	38 (25%)	54 (34%)	.09
Candidiasis, n (%)	155 (50%)	42 (28%)	113 (71%)	**<.01**
Cryptococcosis, n (%)	9 (2%)	0 (0%)	9 (6%)	**<.01**
Toxoplasmosis, n (%)	12 (4%)	0 (0%)	12 (8%)	**<.01**

Data for continuous variables are presented as median and interquartile ranges with *P* values calculated by Kruskal–Wallis test. Data for categorical variables are presented as the absolute count and percentage with *P* values calculated by χ^2^ test.

Abbreviations: ART, antiretroviral therapy; CMV, cytomegalovirus; HSV, herpes simplex virus; IQR, interquartile range; n, number; PcP, *Pneumocystis* pneumonia; PWID, people who inject drugs.

^a^Opportunistic infections data were available for 152 of 239 cases.

### Analytical Sensitivity and Specificity of the Mp1p LFA

Figure 3 *A* shows a representative sample with a gradual dose-response decline in signal intensity by visual reading of the LFA test line corresponding to declining concentrations of rMp1p-spiked urine. Figure 3*B* showed a similar dose-response pattern in cube reader units on rMp1p-spiked urine and plasma samples. Figure 3*C* showed a LoD of 500 pg/mL for both urine and plasma, which is 2-fold higher than the EIA (at 250 pg/mL). No cross-reactivity was observed with 16 clinically related fungi (Supplementary Table 1) or with any of the urine samples from 30 healthy individuals. Only one plasma sample from 30 healthy donors was positive, yielding an overall analytical specificity between 97% and 100%.

**Figure 3.**
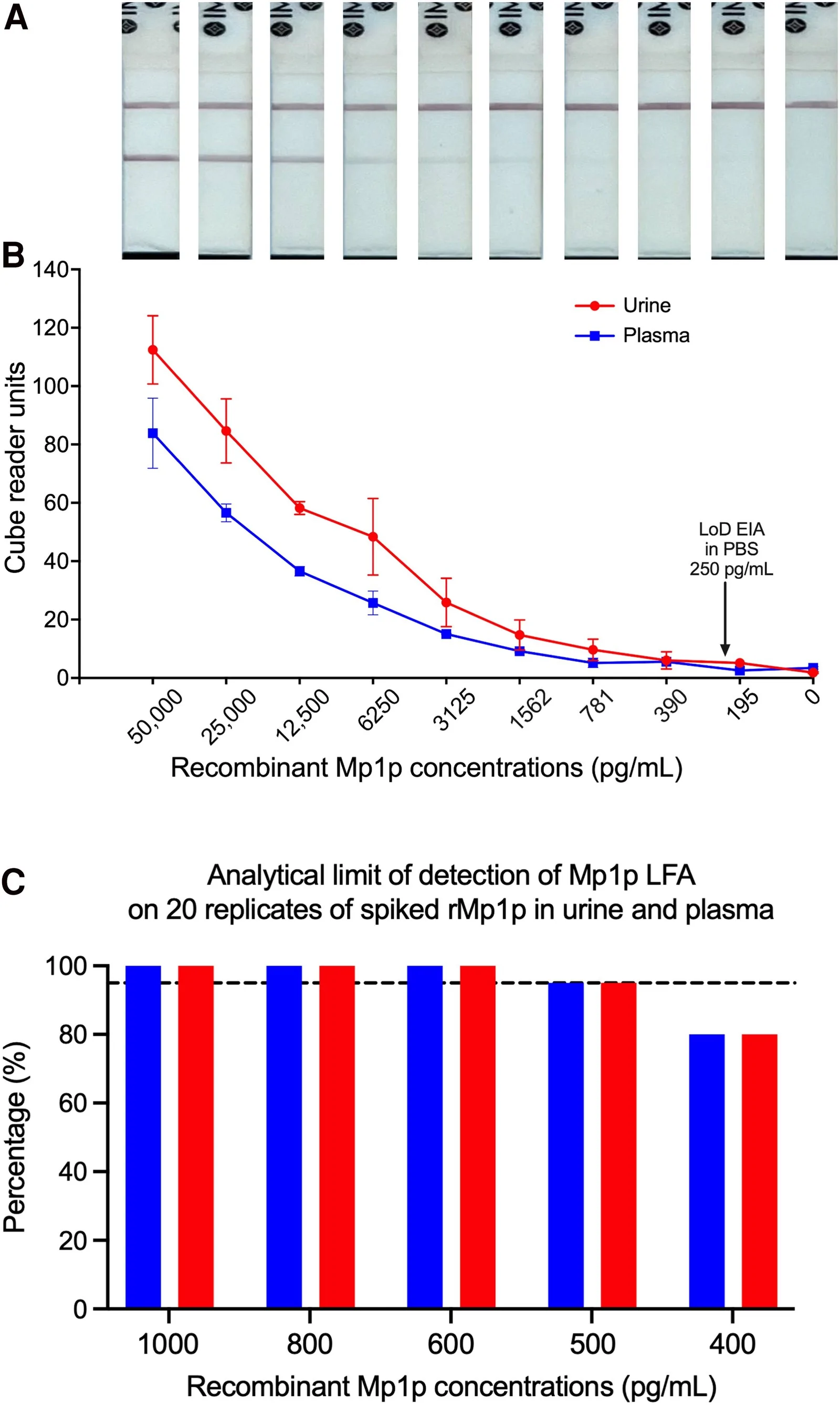
The LoD of the Mp1p LFA. A dose-response reduction in test line intensity was observed in rMp1p-spiked in urine by cube reader (*A*) and in rMp1p-spiked urine and plasma by cube reader reading (*B*). The analytical LoD, defined as the lowest concentration yielding ≥ 95% positive replicates by visual inspection, was 500 pg/mL in urine and plasma (*C*). The LoD of the Mp1p LFA is approximately 2- to 4-fold higher than the LoD of the Mp1p EIA (250 pg/mL). Abbreviations: LFA, lateral flow assay; LoD, limit of detection; PBS, phosphate buffered saline.

### Clinical Performance of the Mp1p LFA by Specimen Type

Figure 4 shows the ROC curves and AUC of the LFA by visual and cube reader readings in the different specimen types. Overall, the discriminative power (ie, the probability of differentiating talaromycosis from non-talaromycosis) was similarly excellent among specimen types (AUC 93.4% to 97.8%). Urine had the best accuracy estimate and was superior to plasma (AUC 96.5% vs 93.4%, *P* < .01). Accuracy estimates were generally similar between visual and cube reader outputs, with urine also having the best accuracy by cube reader output (AUC 97.8%).

**Figure 4.**
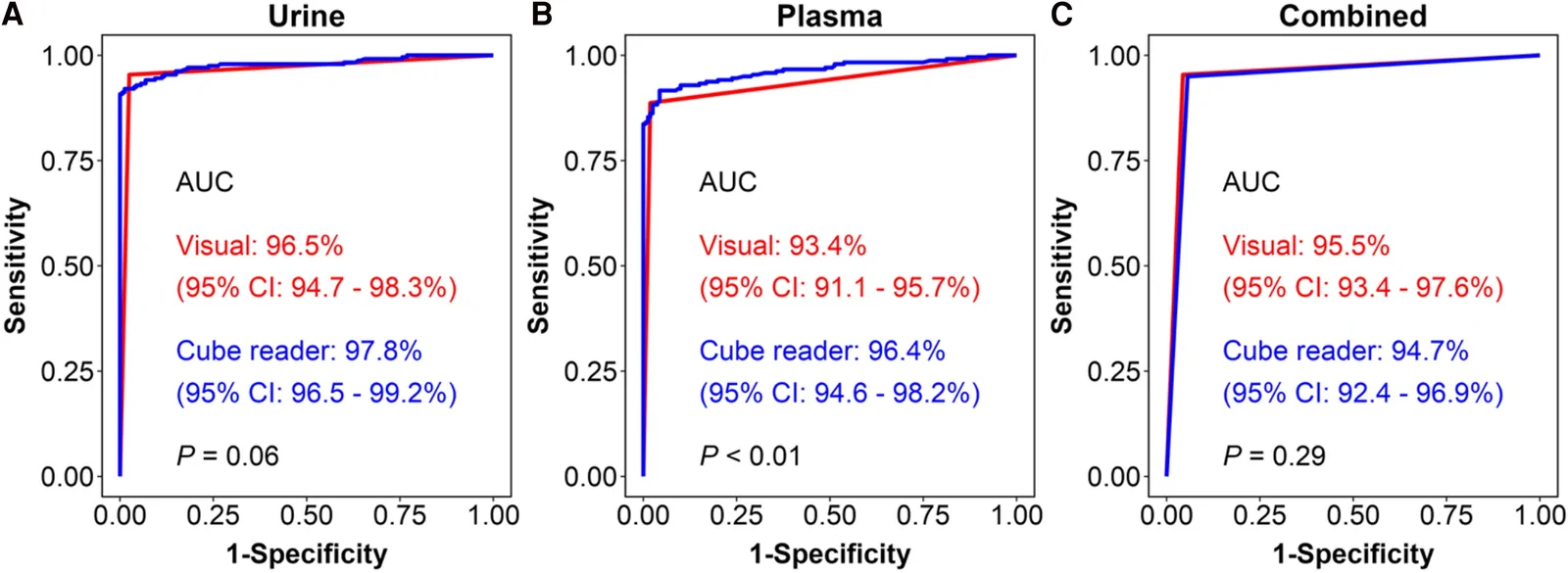
Comparative accuracy of the Mp1p LFA by visual and cube reader readings across specimen types. The discriminative power (AUC values or accuracy) of the Mp1p LFA was similarly excellent among specimen types, and among the 2 reading outputs. Urine had the highest accuracy both by the visual and cube reader readings. Abbreviations: AUC, area under curve; CI, confidence interval; LFA, lateral flow assay.

Table 2 shows the sensitivity, specificity and 95% CI estimates of the LFA by specimen type. Urine demonstrated superior sensitivity compared to plasma (96.5% vs 93.4%, *P* = .03), and all specimen types had high specificity (>95%). Combined testing of urine and plasma did not increase the overall sensitivity (95.4% vs 95.4%), but numerically lowered specificity (95.6% vs 97.5%) compared to testing urine alone.

**Table 2.** Comparative Performance of Mp1p LFA by Visual and Cube Reader Readings in Different Specimen Types

	Mp1p LFA by Visual Reading						*P* Value	Mp1p LFA by Cube Reader						*P* Value
	Urine		Plasma		Combined			Urine		Plasma		Combined		
	Case n = 239	Control n = 160	Case n = 239	Control n = 160	Case n = 239	Control n = 160		Case n = 239	Control n = 160	Case n = 239	Control n = 160	Case n = 239	Control n = 160	
Pos	228	4	212	3	228	7		220	2	219	7	227	9	
Neg	11	156	27	157	11	153		19	158	20	153	12	151	
Cutoffs								6.9		3.6				
AUC (%)	96.5 (94.7–98.3)		93.4 (91.1–95.7)		95.5 (93.4–97.6)			97.8 (96.5–99.2)		96.4 (94.6–98.2)		97.6 (96.1–99.2)		
	Urine versus plasma						***.03***	Urine versus plasma						.22
	Urine versus combined						*.24*	Urine versus combined						**<.01**
	Plasma versus combined						*.12*	Plasma versus combined						.27
Sensitivity (%)	95.4 (91.9–97.7)		88.7 (84.0–92.4)		95.4 (91.9–97.7)		**<.01**	92.1 (87.9–95.1)		91.6 (87.4–94.8)		95.0 (91.4– 97.4)		**.02**
	Urine versus plasma						***<.01***	Urine versus plasma						*1*
	Urine versus combined						*1*	Urine versus combined						.07
	Plasma versus combined						***<.01***	Plasma versus combined						**.04**
Specificity (%)	97.5 (93.7–99.3)		98.1 (94.6–99.6)		95.6 (91.2–98.2)		.16	98.8 (95.6–99.8)		95.6 (91.2–98.2)		94.4 (89.6–97.4)		***.01***
	Urine versus plasma						*1*	Urine versus plasma						*.55*
	Urine versus combined						*.4*	Urine versus combined						*.07*
	Plasma versus combined						*.74*	Plasma versus combined						*1*

Data are presented as point estimates with 95% confidence intervals (CI). Pairwise comparisons of AUC were performed using DeLong's test. The overall difference among the sensitivity and specificity across three specimen types was assessed using Cochran's Q test. Post-hoc pairwise comparisons using McNemar's test. All *P* values of pairwise comparisons were adjusted with Bonferroni correction. Urine demonstrated superior sensitivity and accuracy compared to plasma with the visual reading. Combined testing in urine and plasma did not increase sensitivity beyond testing in urine alone.

Abbreviations: AUC, area under the curve; LFA, lateral flow assay.

### Comparison Between Visual and Cube Reader Outputs of the Mp1p LFA

Supplementary Tables 2 and 3 show the concordance and comparative performance of the LFA when interpreted by visual and cube reader readings. Overall, we observed excellent concordance between visual and cube reader readings for all specimen types (>0.9). In urine, visual reading of the LFA detected a higher proportion of cases compared to cube reader (95.4% vs 92.1%, *P* < .01), whereas both outputs demonstrated similarly high specificity (97.5% vs 98.8%, *P* = .16). In plasma, however, visual reading showed discordance with the cube reader, yielding slightly lower sensitivity (88.7% vs 91.2%, *P* = .02) but higher specificity (98.1% vs 95.6%, *P* = .05). When results from both urine and plasma were combined, the sensitivity and specificity of visual reading were comparable to those obtained using cube reader.

### Comparative Clinical Performance of the Mp1p LFA, the Mp1p EIA, and Blood Culture

The diagnostic performance of the Mp1p EIA (previously performed on the same clinical samples in real-time) is shown in Supplementary Tables 4 and 5. The comparative performance of the LFA, the EIA, and blood culture is shown in Table 3. The LFA provided similar sensitivity (95.4% vs 97.1%, *P* = .87) and specificity (95.6% vs 97.5%, *P* = 1.0) to the EIA. The LR + (ie, probability of a person with the disease testing positive divided by the probability of a person without the disease testing positive) was 21.8 for the LFA and 38.8 for the EIA (*P* = .31). A LR + of 10 or greater indicates a large increase (+45% or higher) in the probability of disease with a positive test. The LR− (ie, the probability of a person with the disease testing negative divided by the probability of a person without the disease testing negative) was 0.05 for the LFA versus 0.03 for the EIA (*P* = .14). A LR− of 0.1 or lower indicates a large decrease (−45% or higher) in the probability of no disease with a negative test. Both the LFA and the EIA were substantially more sensitive than blood culture (95.4% vs 77.8%, *P <* .001 for the LFA, and 97.1% vs 77.8% for the EIA, *P <* .001).

**Table 3.** Comparative Performance of the Mp1p LFA, the Mp1p EIA, and Blood Culture

	Mp1p LFA	Mp1p EIA	Blood Culture	*P* Value
**Sensitivity (%)** (overall)	95.4 (91.9–97.7)	97.1 (94.1–98.8)	77.8 (71.9–82.8)	**<**.**01**
Blood culture positive (n = 186)	97.3 (93.5–99.0)	98.9 (95.8–99.8)	100	.08
Blood culture negative (n = 53)	88.7 (76.3–95.3)	90.6 (78.6–96.5)	…	.65
**Specificity (%)**	95.6 (91.2–98.2)	97.5 (93.7–99.3)	…	1.00
**LR+**	21.8 (10.6–45.0)	38.8 (14.8–102.2)	…	.31
**LR−**	0.05 (0.03–0.09)	0.03 (0.01–0.06)	…	.14

Data are presented as percentages (%) with 95% confidence intervals. Diagnostic performance of the Mp1p LFA and the Mp1p EIA in combined testing of urine and plasma. The Mp1p LFA was interpreted by visual inspection. Both the Mp1p LFA and the Mp1p EIA provided substantially higher sensitivity than blood culture.

Abbreviations: EIA, enzyme immunoassay; LFA, lateral flow assay; LR+, positive likelihood ratio; LR−, negative likelihood ratio.

### Comparison of the Quantitative Outputs of the Mp1p LFA Versus the Mp1p EIA

Figure 5 displays the distribution cube reader units of the LFA and the OD values of the EIA in urine and plasma among cases and controls and their corresponding ROC curves. The cube reader units generated by the LFA followed a similar distribution to the OD values generated from the EIA. For the LFA, the Youden-index-generated cube reader cutoff was higher in urine (6.9) than in plasma (3.6) for this data set and were close to the preliminary reader cutoff (5). We observed excellent concordance between the quantitative outputs of the LFA cube reader and the EIA across all specimen types (0.91 in urine, 0.88 plasma, and 0.91 in combined testing of urine and plasma (Supplementary Table 6).

**Figure 5.**
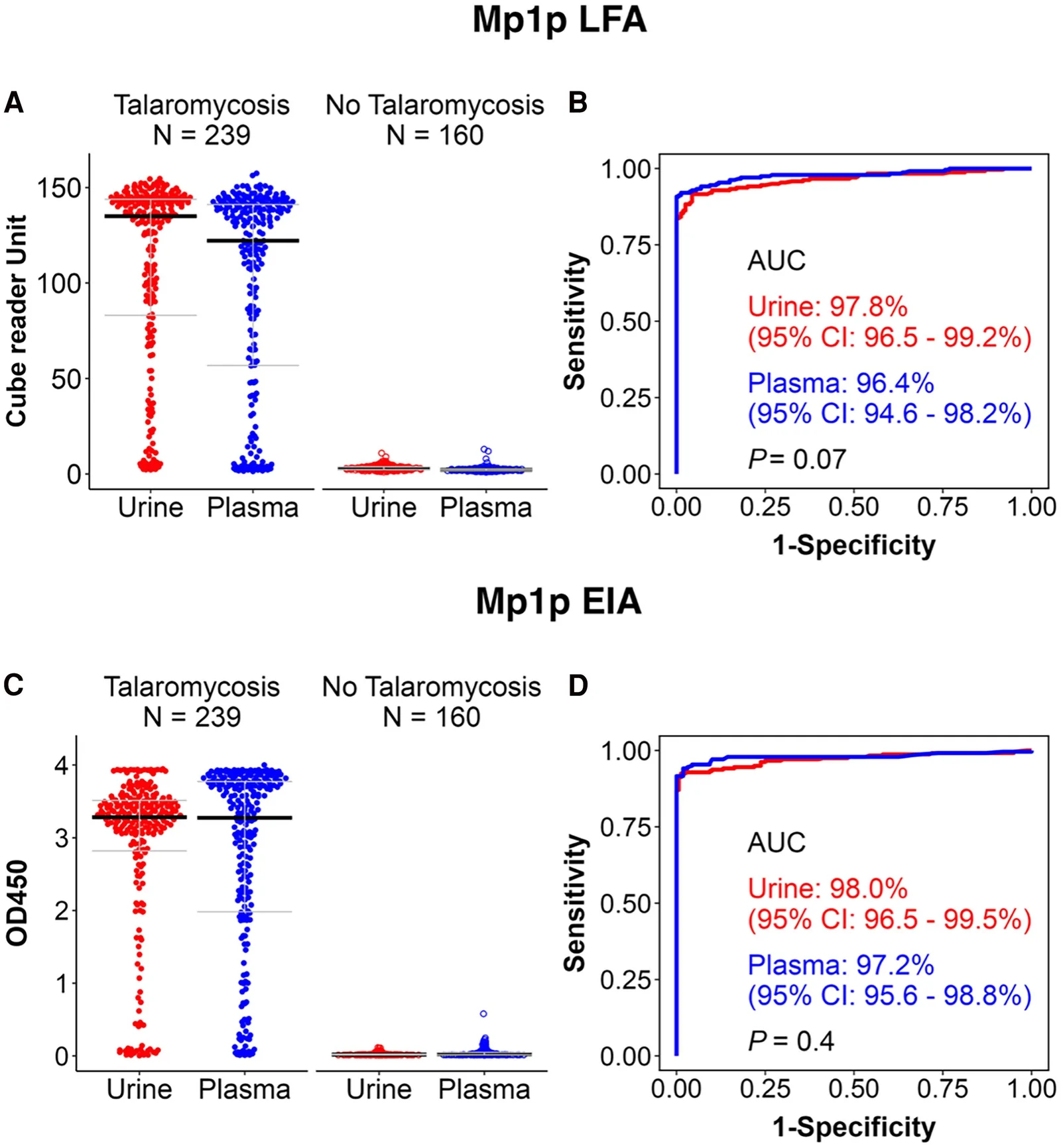
Comparison of the quantitative outputs of the Mp1p LFA cube reader versus the Mp1p EIA spectrophotometer. The cube reader units generated by Mp1p LFA (*A*) followed a similar distribution to the optical density values generated by the Mp1p EIA (*C*). The discriminative power was similar in urine (red) versus plasma (blue) for the Mp1p LFA (*B*) and for the Mp1p EIA (*D*). Abbreviations: AUC, area under curve; CI, confidence interval; EIA, enzyme immunoassay; LFA, lateral flow assay; OD450, optical density at a wavelength of 450 nanometers; ROC, receiver operating characteristic.

### Evaluation of the False Positive and False Negative Cases by Visual LFA Results

There were 11 false negative and 7 false positive LFA results compared to the reference standard. Among the 11 false negatives, 6 (55%) were diagnosed from non-blood specimens, and 7 (64%) had negative EIA results, suggesting that false negative result is associated with low fungal burden in the blood. The remaining 5 false negatives had positive blood culture, and none had evidence of post-zone effect. There was no noticeable difference in the distribution of the 11 false negatives with respect to phylogenetic clade or geographical location. The 7 false positives had no clustering of infections to suggest cross-reactivity with specific pathogens. Of the 7 false positive LFA results, EIA was also positive in 1 case, showing low positive OD in plasma and low cube reader values in plasma and urine, possibly indicating that this was in fact a true positive in a patient with low fungal burdens that was missed by culture methods.

## DISCUSSION

We developed a sensitive and highly specific Mp1p LFA for the rapid diagnosis of talaromycosis. Our study also represents the largest clinical evaluation of a point-of-care test for talaromycosis to date [11]. In our case-cohort study of 399 patients with AHD, the overall sensitivity and specificity of the LFA were excellent (sensitivity 95%, specificity 96%), matching that of the EIA in head-to-head comparison. Similar to the EIA, the LFA offered superior sensitivity to blood culture tested from the same participants in the same body compartment (78%), demonstrating its value in making an accurate and rapid diagnosis of talaromycosis, potentially cutting down the time-to-diagnosis by 4–28 days [11]. The LFA demonstrated a high LR + (>10) and low LR− (<0.1), suggesting promising utility as a rapid rule-in and rule-out test for talaromycosis in hospitalized patients with AHD.

Remarkably, the Mp1p LFA performed the best in urine, providing higher sensitivity than plasma without sacrificing specificity. However, unlike the case for the EIA performed on the same clinical samples, combined testing of urine and plasma did not improve sensitivity but numerically reduced specificity in this validation cohort. Our data showed that urine is a superior specimen for Mp1p LFA testing. Urine is also a noninvasive and a relatively easier specimen to collect than blood in clinic and community settings, and is therefore our recommended specimen type for LFA testing.

The LFA was primarily developed as a qualitative assay, but we have shown it is possible to derive semi-quantitative antigen levels using commercially available portable readers, which would remove visual variability and ambiguity, while allowing for semi-quantification of antigen that may have utility in disease prognostication and monitoring. Although the discordance between visual inspection and cube reader interpretation was marginal and unlikely to meaningfully affect post-test probability, further studies are needed to evaluate the reproducibility, reliability, and feasibility of cube reader for point-of-care testing. Mp1p antigenemia (detected by EIA) has been shown to precede blood culture positivity by up to 16 weeks, indicating utility in identifying early, subclinical infections [19]. The lower limit of detection of the Mp1p EIA and its ability to quantify antigen levels may prove more suitable for detection of patients with very low antigenemia. Future studies are needed to determine if the Mp1p LFA can be used to screen patients with AHD before the onset of clinical symptoms [35]

Our study has some limitations and strengths. First, there were some differences in the characteristics of the cases and controls including sex, intravenous drug use, median CD4 counts and rates of tuberculosis and bacterial sepsis, which may affect assay performance. However, both our case and control populations consisted of clinically relevant patients with AHD (CD4 < 50 cells/μL) with a range of other opportunistic infections, who are representative of the population at risk and who would be tested for talaromycosis in real life [2]. Second, the LFA was performed on archived specimens collected between 2012 and 2018, whereas the EIA was performed in real time as part of a prospective validation study. There exists the potential for antigen degradation during storage which may have underestimated the sensitivity of the LFA compared to the EIA. Third, we were unable to explain some of the false negative and false positive results beyond that the fact the LFA has slightly higher LoD compared to the EIA, and false negatives were most likely due to low fungal burden. Finally, our evaluation was limited to individuals with AHD in Vietnam. Given the evolving epidemiology of talaromycosis [5,6, 36, 37], the Mp1p LFA should be further evaluated in individuals with immunocompromising disorders other than human immunodeficiency virus (HIV) and in returning travelers in non-endemic settings as performance may vary across these distinct populations.

## CONCLUSIONS

The Mp1p LFA demonstrated excellent diagnostic performance and the potential to rapidly rule in and rule out talaromycosis in 15 minutes at the point-of-care. Future prospective evaluations should determine its clinical utility in making a rapid diagnosis, disease prognostication, and in a screen and treat strategy to prevent talaromycosis.

## Supplementary Material

ciag004_Supplementary_Data

## Supplementary Data

Supplementary materials are available at *Clinical Infectious Diseases* online. Consisting of data provided by the authors to benefit the reader, the posted materials are not copyedited and are the sole responsibility of the authors, so questions or comments should be addressed to the corresponding author.

## Notes

***Author contributions.*** Study concept and design: T. L., K. B., O. H. Obtaining funding: T. L., K. B. Contributing raw materials and technical expertise: K. B., O. H. Contributing to patient recruitment, patient samples, and clinical expertise: V. T. L. and T. L. Laboratory work: K. B., O. H., M. B., J. F., N. T. M. T., S. V., H. N. S., L. B. Data analyses: N. T. M. T., O. H., S. V., L. B., T. L., K. B. Interpretation of data: N. T. M. T., L. B., S. V., M. B., N. T. H., T. L. Drafting the manuscript: N. T. M. T., L. B., S. V. Critical revision of the manuscript for intellectual content: All authors. All authors contributed to and approved the final manuscript.

***Acknowledgments.*** The authors thank all participants, investigators and staff who participated in this study across Vietnam, and supporting staff from the Clinical Trials Units at the Oxford University Clinical Research Unit in Vietnam for the coordination of the clinical studies. They are grateful to researchers at the University of Hong Kong who provided access to the original raw materials including Mp1p antigen, protein and antibody sequences, and technical expertise, and to researchers at Duke University who provided technical and clinical expertise in the study design and implementation.

***Ethical statement.*** This diagnostic evaluation utilized archived plasma and urine samples collected as a sub-study of the multi-center Itraconazole versus Amphotericin B for Penicilliosis (IVAP) randomized controlled trial [7] and a single-center, prospective cohort study [31]. All participants gave informed consent for specimens to be stored and used in this research. The sub-study was approved by local hospitals in Vietnam and at Duke University.

***Financial support.*** This work was supported by the National Institutes of Health (NIH) (grant numbers R01AI143409 and U01AI169358 to T. L.). The Mp1p lateral flow assay (LFA) kits were developed by Immuno-Mycologics, Inc. (**I**MMY, Norman, Oklahoma, USA) with the support of the NIH (contract number 75N93022C00027). The NIH had no role in the design and conduct of the study.
