# Supplementary material for: Development and Clinical Evaluation of a Novel Talaromyces marneffei Mp1p Lateral Flow Assay for Rapid Diagnosis of Talaromycosis
Source: Clin Infect Dis. 2026 Jan 6;83(3):e495–504. doi: 10.1093/cid/ciag004 (PMC13644868; doi:10.1093/cid/ciag004)
Supplement: Supplementary file 1 [file ciag004_supplementary_data.docx]

**Supplementary Materials:** Development and Clinical Evaluation of a Novel *Talaromyces marneffei* Mp1p Lateral Flow Assay for Rapid Diagnosis of Talaromycosis

**Authors:** Nguyen Thi Mai Thu^1,8*^, Lottie Brown^2,3*^, Oriana Hawkins^4^, Konner Bloss^4^, Sruthi Venugopalan^5^, Meredith Brown^4^, Jonathan Fenhaus^4^, Heera Natesan Sambath^1^, Jialin Liu^1^, Lyla Clancy^1^, Jian-Piao Cai^6^, Jasper Fuk-Woo Chan^6,7^, KY Yuen^5,6^, Vo Trieu Ly^8,7^, Ngo Thi Hoa^9,10,11^, Thuy Le^1,9^

**Affiliations:**

^1^ Division of Infectious Diseases and International Health, Duke University School of Medicine, Durham, NC, USA

^2^ Clinical Infection Unit, St George’s Hospital, St George’s NHS Foundation Trust, London, UK

^3^ Institute of Infection and Immunity, School of Health and Medical Sciences, City St George’s University of London, London, UK

^4^ Immuno-Mycologics, Inc. (IMMY), Norman, OK, USA

^5^ Department of Internal Medicine, University of Nevada Reno School of Medicine, Reno, Nevada, USA

^6^ State Key Laboratory of Emerging Infectious Diseases, Carol Yu Center for Infection, Department of Microbiology, School of Clinical Medicine, Li Ka Shing Faculty of Medicine, the University of Hong Kong, Pokfulam, Hong Kong Special Administrative Region, China

^7^ Department of Infectious Diseases and Microbiology, The University of Hong Kong-Shenzhen Hospital, Shenzhen, Guangdong Province, China

^8^ Hospital for Tropical Diseases, Ho Chi Minh City, Vietnam

^9^ Tropical Medicine Research Center for Talaromycosis, Centre for Biomedical Research, Pham Ngoc Thach University of Medicine, Ho Chi Minh City, Vietnam

^10^ Oxford University Clinical Research Unit, Ho Chi Minh City, Vietnam

^11^ Nuffield Department of Medicine, University of Oxford, Oxford, United Kingdom

*^*^* These authors contributed equally to this work

**Corresponding Authors:**

Lottie Brown, MD, MSc, Institute of Infection and Immunity, School of Health and Medical Sciences, City St George’s Hospital and St George’s University, Cranmer Terrace, London, UK, SW17 0RE. Phone: 020 7040 5060. Email: [lotbrown@sgul.ac.uk](mailto:lotbrown@sgul.ac.uk)

Thuy Le, MD, PhD, Associate Professor of Medicine. Division of Infectious Diseases and International Health, Duke University School of Medicine. 315 Trent Drive, Office #257, Durham, North Carolina, USA, 27710. Phone: 919.668.5053. Fax: 919.681.8902. Email: [thuy.le@duke.edu](mailto:thuy.le@duke.edu)

**Supplementary Figure S1: The Workflow of the Mp1p LFA**

A summary of the workflow for the Mp1p LFA. To perform the assay, 60 μL of specimen diluent buffer was added to 40 μL of plasma or urine in a 2 mL flat bottom microcentrifuge tube and mixed well. The Mp1p LFA strip was placed in the diluted specimen and incubated at room temperature for 15 minutes. The presence of two bands (a control band and a test band) in the test zone of the strip was interpreted as a positive result and a single band in the test zone (a control band) was interpreted as a negative result.

Abbreviations: μL, microlitre; mL, millilitre; mins, minutes.

**Supplementary Figure S2: Diagnostic reference standard for clinical evaluation**

In line with the European Organization for Research and Treatment of Cancer and the Mycoses Study Group Education and Research Consortium (EORTC/MSGERC) definitions of *Clinical Infectious Diseases.* proven endemic mycoses^*^, talaromycosis was defined as a compatible clinical syndrome plus *T. marneffei-*positive culture of blood, skin lesions, lymph nodes, bone marrow, or other sterile sites, tested as clinically indicated.

* Donnelly et al. *Clinical Infectious Diseases.* 2020;15;71(6):1367-76.

**Supplementary Figure S3: Sample Size Calculations**

Sample size calculations were based on previously reported sensitivity (86%) and specificity (96%) of our previously-validated Mp1p EIA and used the Hajian-Tilaki’s method for diagnostic test studies which takes into account both sensitivity and specificity. A sample size of 239 cases and 160 controls would provide a power of 80% or higher to detect a sensitivity of 90% (error d = 0.07) and a specificity of 96% (error d = 0.04) with an α = 0.05 for the Mp1p EIA.

Sample size calculations were performed using the method of Hajian-Tilaki (*J Biomed Inform*. 2014;48:193–204).

Abbreviations: EIA, enzyme immunoassay.

**Supplementary Table S1.** **Cross-reactivity of the Mp1p LFA with clinically related fungal isolates**.

| Culture filtrates | Dilution | | | |
| --- | --- | --- | --- | --- |
|  | Neat | 1:10 | 1:100 | 1:1000 |
| *Aspergillus flavus* | - | - | - | - |
| *Aspergillus fumigatus* | - | - | - | - |
| *Aspergillus Niger* | - | - | - | - |
| *Aspergillus Terreus* | - | - | - | - |
| *Candida albicans* | - | - | - | - |
| *Histoplasma capsulatum* Mycelium | - | - | - | - |
| *Histoplasma* *capsulatum* Yeast | - | - | - | - |
| *Blastomyces dermatidis* | - | - | - | - |
| Antigens | Dilution | | | |
|  | 50 µg/mL | 5 µg/mL | 0.5 µg/mL | 0.05 µg/mL |
| *Cryptococcus* Serotype A, F184A | - | - | - | - |
| *Cryptococcus* Serotype A, CDC B551 | - | - | - | - |
| *Cryptococcus* Serotype B, H381B | - | - | - | - |
| *Cryptococcus* Serotype B, 409 | - | - | - | - |
| *Cryptococcus* Serotype C, B187C | - | - | - | - |
| *Cryptococcus* Serotype C, KC34 | - | - | - | - |
| *Cryptococcus* Serotype D, B3501D | - | - | - | - |
| *Cryptococcus* Serotype D, EJ-127 | - | - | - | - |

Results are presented as visual readings of 40µL of ten-fold serial dilutions (10^3^ – neat) for culture filtrates and 0.05 – 50 µg/mL for antigens.

Abbreviations: μg, microgram; mL, millilitre.

**Supplementary Table S2: Concordance of the Mp1p LFA results when interpreted by visual inspection and by cube reader.**

|  | **Cases (n = 239)** | | | **Controls (n = 160)** | | | $\mathbf{K}$ |
| --- | --- | --- | --- | --- | --- | --- | --- |
|  | Mp1p LFA positive (Visual) | Mp1p LFA negative (Visual) | Sum row | Mp1p LFA positive (Visual) | Mp1p LFA negative (Visual) | Sum row |  |
| **Urine** |  |  |  |  |  |  | 0.95 |
| Mp1p LFA positive (Cube reader) | 220 | 8 | 228 | 2 | 2 | 4 |  |
| Mp1p LFA negative (Cube reader) | 0 | 11 | 11 | 0 | 156 | 156 |  |
| Sum column | 220 | 19 | 239 | 2 | 158 | 160 |  |
| **Plasma** |  |  |  |  |  |  | 0.93 |
| Mp1p LFA positive (Cube reader) | 211 | 1 | 212 | 3 | 0 | 3 |  |
| Mp1p LFA negative (Cube reader) | 8 | 19 | 27 | 4 | 153 | 157 |  |
| Sum column | 219 | 20 | 239 | 7 | 153 | 160 |  |
| **Combined** |  |  |  |  |  |  | 0.91 |
| Mp1p LFA positive (Cube reader) | 227 | 0 | 227 | 5 | 4 | 9 |  |
| Mp1p LFA negative (Cube reader) | 1 | 11 | 12 | 2 | 149 | 151 |  |
| Sum column | 228 | 11 | 239 | 7 | 153 | 160 |  |

Cohen’s kappa coefficient ($K$) measures the level of agreement between paired binary variables. Interpretation of kappa values followed by < 0.20 (poor), 0.21 – 0.40 (fair), 0.41 – 0.60 (moderate), 0.61 – 0.80 (substantial), > 0.81 (almost perfect agreement).

Abbreviations: EIA, enzyme immunoassay; LFA, lateral flow assay; n, number.

**Supplementary Table S3: Comparative performance of the Mp1p LFA when interpreted by visual inspection and by cube reader.**

|  | **Mp1p LFA by visual readings** | **Mp1p LFA by cube reader units** | ***P* values** |
| --- | --- | --- | --- |
| **Urine** |  |  |  |
| Sensitivity (%) | 95.4 (91.9 – 97.7) | 92.1 (87.9 - 95.1) | **<0.01** |
| Specificity (%) | 97.5 (93.7 – 99.3) | 98.8 (95.6 - 99.8) | 0.16 |
| LR+ | 38.2 (14.5 – 100.5) | 73.6 (18.6 - 292.0) | 0.19 |
| LR- | 0.05 (0.03 – 0.08) | 0.08 (0.05 - 0.12) | **<0.01** |
| **Plasma** |  |  |  |
| Sensitivity (%) | 88.7 (84.0 – 92.4) | 91.2% (86.9 - 94.5%) | **0.02** |
| Specificity (%) | 98.1 (94.6 – 99.6) | 95.6% (91.1 - 98.2%) | **0.046** |
| LR+ | 47.3 (15.4 – 145.3) | 20.8 (10.1 - 43.1) | 0.06 |
| LR- | 0.11 (0.08 – 0.16) | 0.09 (0.06 - 0.14) | **0.03** |
| **Combined testing in plasma and urine** | |  |  |
| Sensitivity (%) | 95.4 (91.9 – 97.7) | 95.0% (91.4 - 97.4%) | 0.32 |
| Specificity (%) | 95.6 (91.2 – 98.2) | 94.4% (89.6 - 97.4%) | 0.41 |
| LR+ | 21.8 (10.6 – 45.0) | 16.9 (8.9 - 31.9) | 0.41 |
| LR- | 0.05 (0.03 – 0.09) | 0.05 (0.03 - 0.09) | 0.26 |

Statistical significance of the difference in sensitivity and specificity was assessed using McNemar’s test and in likelihood ratios using regression model approach. LR+ is undefined when specificity = 100%.

Abbreviations: LFA, lateral flow assay; LR+, positive likelihood ratio; LR-, negative likelihood ratio; n, number.

**Supplementary table S4. Diagnostic performance of the Mp1p LFA (interpreted visually) and the Mp1p EIA across specimen types**

|  | **Urine** | | **Plasma** | | **Urine and/or plasma** | |
| --- | --- | --- | --- | --- | --- | --- |
|  | Cases  (n = 239) | Controls  (n = 160) | Cases  (n = 239) | Controls  (n = 160) | Cases  (n = 239) | Controls  (n = 160) |
| **Mp1p LFA** |  |  |  |  |  |  |
| Positive | 228 | 4 | 212 | 3 | 228 | 7 |
| Negative | 11 | 156 | 27 | 157 | 11 | 153 |
|  |  |  |  |  |  |  |
| Sensitivity (%) | 95.4 (91.9 – 97.7) | | 88.7 (84.0 – 92.4) | | 95.4 (91.9 – 97.7) | |
| Specificity (%) | 97.5 (93.7 – 99.3) | | 98.1 (94.6 – 99.6) | | 95.6 (91.2 – 98.2) | |
| LR+ | 38.2 (14.5 – 100.5) | | 47.3 (15.4 – 145.3) | | 21.8 (10.6 – 45.0) | |
| LR- | 0.05 (0.03 – 0.08) | | 0.12 (0.08 – 0.16) | | 0.05 (0.03 – 0.09) | |
| DOR | 808.4 (252.8 – 2584.7) | | 410.9 (122.5 – 1378.7) | | 453.0 (171.8 – 1194.5) | |
|  |  | |  | |  | |
| **Mp1p EIA** |  | |  | |  | |
| Positive | 225 | 3 | 221 | 2 | 232 | 4 |
| Negative | 14 | 157 | 18 | 158 | 7 | 156 |
|  |  | |  | |  | |
| Sensitivity (%) | 94.1 (90.4 – 96.8) | | 92.5 (88.4 – 95.5) | | 97.1 (94.1 – 98.8) | |
| Specificity (%) | 98.1 (94.6 – 99.6) | | 98.8 (95.6 – 99.8) | | 97.5 (93.7 – 99.3 ) | |
| LR+ | 50.2 (16.4 – 154.1) | | 74.0 (18.7 – 293.4) | | 38.8 (14.8 – 102.2) | |
| LR- | 0.06 (0.04 – 0.10) | | 0.08 (0.05 – 0.12) | | 0.03 (0.01 – 0.06) | |
| DOR | 841.1 (237.7 – 2975.5) | | 969.9 (221.9 – 4240.0) | | 1292.6 (372.1 – 4489.6) | |

Data are presented as point estimates with 95% confidence intervals (CI). Diagnostics odds ratio (DOR) is a measure of overall diagnostic accuracy incorporating both sensitivity and specificity.

Abbreviations: EIA, enzyme immunoassay; LFA, lateral flow assay; LR+, positive likelihood ratio; LR-, negative likelihood ratio; DOR, Diagnostic Odds ratio.

|  | Urine | Plasma | Combined Testing in Urine and Plasma | *P* value |
| --- | --- | --- | --- | --- |
| Sensitivity (%) | 94.1 (90.4 – 96.8) | 92.5 (88.4 – 95.5) | 97.1 (94.1 – 98.8) | **< 0.01** |
| Urine *vs* plasma | | | | 1.00 |
| Urine *vs* combined testing | | | | 0.07 |
| Plasma *vs* combined testing | | | | **< 0.01** |
| Specificity (%) | 98.1 (94.6 – 99.6) | 98.8 (95.6 – 99.8) | 97.5 (93.7 – 99.3) | 0.37 |
| LR+ | 50.2 (16.4 – 154.1) | 74.0 (18.7 – 293.4) | 38.8 (14.8 – 102.2) |  |
| Urine *vs* plasma | | | | 0.58 |
| Urine *vs* combined testing | | | | 0.89 |
| Plasma *vs* combined testing | | | | 0.20 |
| LR- | 0.06 (0.04 – 0.10) | 0.08 (0.05 – 0.12) | 0.03 (0.01 – 0.06) |  |
| Urine *vs* plasma | | | | 0.36 |
| Urine *vs* combined testing | | | | **0.01** |
| Plasma *vs* combined testing | | | | **< 0.01** |

**Supplementary table S5. Comparative diagnostic performance of the Mp1p EIA on different specimen types**

Differences between specimen types were evaluated by Cochran’s Q test for paired binary data. When a statistically significant difference was detected, post-hoc pairwise comparisons were performed using McNemar’s test adjusted with Ferroboni correction.

Abbreviations: LR+, positive likelihood ratio; LR-, negative likelihood ratio.

**Supplementary Table S6: Concordance of Mp1p LFA results (by visual reading) and Mp1p EIA results**

|  | **Cases (n = 239)** | | | **Controls (n = 160)** | | | $\mathbf{K}$ |
| --- | --- | --- | --- | --- | --- | --- | --- |
|  | Mp1p LFA positive | Mp1p LFA negative | Sum row | Mp1p LFA positive | Mp1p LFA negative | Sum row |  |
| **Urine** |  |  |  |  |  |  | 0.91 |
| Mp1p EIA positive | 221 | 4 | 225 | 0 | 3 | 3 |  |
| Mp1p EIA negative | 7 | 7 | 14 | 4 | 153 | 157 |  |
| Sum column | 228 | 11 | 239 | 4 | 156 | 160 |  |
| **Plasma** |  |  |  |  |  |  | 0.88 |
| Mp1p EIA positive | 207 | 14 | 221 | 0 | 2 | 2 |  |
| Mp1p EIA negative | 5 | 13 | 18 | 3 | 155 | 158 |  |
| Sum column | 212 | 27 | 239 | 3 | 157 | 160 |  |
| **Combined** |  |  |  |  |  |  | 0.91 |
| Mp1p EIA positive | 226 | 6 | 232 | 1 | 3 | 4 |  |
| Mp1p EIA negative | 2 | 5 | 7 | 6 | 150 | 156 |  |
| Sum column | 228 | 11 | 239 | 7 | 153 | 160 |  |

Mp1p LFA result determined by visual reading. Cohen’s kappa coefficient ($K$) measures the level of agreement between paired binary variables. Interpretation of kappa values followed by < 0.20 (poor), 0.21 – 0.40 (fair), 0.41 – 0.60 (moderate), 0.61 – 0.80 (substantial), > 0.81 (almost perfect agreement).

Abbreviations: EIA, enzyme immunoassay; LFA, lateral flow assay; n, number.
